# How Normal Is the Liver in Which the Inflammatory Type Hepatocellular Adenoma Develops?

**DOI:** 10.1155/2012/805621

**Published:** 2012-09-17

**Authors:** Jing Han, Marius C. van den Heuvel, Hironori Kusano, Koert P. de Jong, Annette S. H. Gouw

**Affiliations:** ^1^Department of Pathology and Medical Biology, University Medical Center Groningen, Hanzeplein 1, P.O. Box 30.001, 9700 RB Groningen, The Netherlands; ^2^Pathology Laboratory Friesland, Postbus 3305, 8901 DH, Leeuwarden, The Netherlands; ^3^Division of Hepatobiliary Surgery and Liver Transplantation, Department of Surgery, University Medical Center Groningen, Hanzeplein 1, P.O. Box 30.001, 9700 RB Groningen, The Netherlands

## Abstract

The inflammatory type hepatocellular adenoma (IHCA) is a subtype of HCA which is a benign liver tumor, predominantly occurring in young women in an otherwise normal liver. IHCA contains either a mutation of gp130 or STAT3. Both mutations lead to a similar morphologic phenotype and to increased expression of C-reactive protein (CRP) and/or serum amyloid-A (SAA). IHCA comprised about 40% of all HCAs and is associated with obesity. We investigated the histomorphological and immunophenotypical changes of the nontumorous liver of 32 resected IHCA specimens. Similar types of changes are present in samples taken adjacent to tumor and distant ones. The lobular architecture is well preserved. Mild/moderate steatosis is found in a high frequency which is in accordance with the median BMI of 32 in our cases. Of note are the regular findings of sinusoidal dilatation, single arteries, and minute CRP foci which are all features of HCA. These distinct CRP foci are mostly found in cases of multiple IHCA which indicates that the remnant liver may also contain IHCA foci. These findings show that the nonlesional liver in IHCA does contain abnormalities, and this may have consequences for the followup, especially since it is known that obesity may stimulate malignant growth.

## 1. Introduction 

Hepatocellular adenoma (HCA) is a benign primary hepatocellular tumor, occurring predominantly in females in their reproductive age and is associated with long-term use of oral contraceptives [1, 2]. Recently, a rising incidence has been reported, partly due to improved application of diagnostic imaging techniques, for example, CT, MRI [3]. HCA is divided into 3 subgroups according to 3 different genetic mutations: hepatocellular nuclear factor-1*α* (HNF1*α*) gene-mutated type HCA, *β*-catenin gene-mutated type HCA, and inflammatory type HCA (IHCA) which contains a somatic mutation of IL6ST gene. The latter mutation, encoding gp130, is found in 60% of IHCA, and a somatic mutation of STAT3 gene is found in 12% of IHCA [4, 5]. A fourth group represents HCA without any of these mutations. Of note, the IHCA may concurrently contain *β*-catenin mutation which increases the risk of malignant transformation. The HCA subtyping can be performed by visualizing the coded proteins of the mutated genes by immunohistology [6–8]. HCA containing HNF1*α* mutation shows absence of liver fatty acid binding protein-1 (LFABP-1) in contrast with the diffuse hepatocytic expression of this protein in normal livers. IHCAs, both those with IL6ST mutation and STAT3 gene mutation, show increased C-reactive protein (CRP) and/or serum amyloid-A (SAA) expression [5]. HCA containing *β*-catenin mutation shows nuclear translocation of *β*-catenin expression, but this finding may be focal and patchy, whereas an aberrant diffuse expression of glutamine synthetase (GS) is also indicative of *β*-catenin mutation [6–8]. 

IHCA represents the largest subgroup of HCA and has been reported to be related with systemic disorders, such as obesity, metabolic syndrome, and alcohol abuse [7, 9]. One report mentioned that IHCA patients with a high body mass index (BMI ≥ 25) represent 60% of their study group in which the mean BMI is 28 [9]. Subgroups of HCA except the *β*-catenin gene-mutated type rarely show malignant transformation into hepatocellular carcinoma (HCC) although a recent study reported an increased risk in HCA occurring in overweight or obese male patients [10]. These findings suggest that in obese individuals the whole hepatic microenvironment is influenced by systemic factors that may favor tumor development, in accordance with the postulation that obesity increases the risk of cancer development [11].

In the present study, we investigated the histological features of the nontumorous liver parts of 32 resected IHCA specimens, to gain insight in the hepatic microenvironment in which IHCA develops, also because IHCA are often multifocal. Therefore, knowledge about the nonlesional liver tissue that corresponds to the remnant liver after tumor resection may influence the followup management of IHCA patients.

We found that although the lobular architecture is largely well preserved, the nontumorous liver frequently shares several abnormal features with the adenoma, such as sinusoidal dilatation and single arteries. Moreover, many cases also contain several foci of minute HCA-like areas with focal increase of CRP/SAA. These findings suggest that the nonlesional part of HCA-containing livers harbors changes that may potentially stimulate adenomatous growth. This is especially true for livers with multiple adenomas.

## 2. Patients and Methods

### 2.1. Patients

Thirty two patients, all of them females (mean age 33.5 ± 8.8 years), who underwent partial liver resection for IHCA, were included. Cases were selected on the availability of sufficient amount of adjacent nontumorous liver (AL) and/or distant nonlesional liver tissue (DL). The latter sample was taken at least 3 cm distant from the tumor. 

### 2.2. Histology

A representative slide of the transformation area of tumor and adjacent nontumorous liver tissue (AL, *n* = 32) and one sample from the distant nonlesional part (DL, *n* = 22) were reviewed without knowledge of clinical data and the features of the corresponding tumor. Slides were stained with hematoxylin-eosin (HE) and Masson trichrome. The AL and DL samples were assessed separately for the following features: liver architecture, steatosis, steatohepatitis, sinusoidal dilation, single artery, and ductular reaction. Grading of steatosis and steatohepatitis was performed according to the scoring system for nonalcoholic steatohepatitis (NASH) proposed by Brunt et al. [12]. In summary, steatosis: 0 = absent; 1 = steatosis observed in up to 33%; 2 = more than 33% and less than 66%; 3 = more than or equal to 66% and steatohepatitis: 0 = absent; 1 = occasional ballooned hepatocytes, mild portal chronic inflammation; 2 = obvious ballooned hepatocytes, portal and intra-acinar chronic inflammation noted, mild to moderate; 3 = ballooning and disarray obvious with mild chronic inflammation, portal chronic inflammation, mild or moderate. 

Grading of sinusoidal dilation followed the criteria mentioned by Rubbia-Brandt et al. [13]. Sinusoidal dilation: 0 = absent; 1 = centrilobular involvement limited to one-third of lobular surface; 2 = two-thirds lobular surface involved; 3 = complete lobular surface involved. Liver architecture is scored as preserved (1) or abnormal (0). Single artery and ductular reactions (DRs) are described as absent (0) or present (1). Single arteries are defined as arterial structures without accompanying bile duct and/or not localized in a portal tract structure. Assessment of DR is described below.

### 2.3. Immunohistochemistry

The immunohistological expression of SAA/CRP on tumor tissue was already performed at an earlier, diagnostic stage to establish the diagnosis of IHCA according to the Bordeaux classification [7]. GS and *β*-catenin staining were also completed at the earlier diagnostic stage to assess possible *β*-catenin mutation. For the present study, AL and DL samples were stained according to the same protocol, and additional immunostaining with K19, CD34, and *α*-SMA was performed. K19 increased the feasibility to assess DR as the ductular structures were highlighted by K19 labeling. The presence of 4 or more ductular profiles per portal tract is regarded as the presence of DR [14]. 

CD34 visualized sinusoidal capillarization and single arteries, whereas *α*-SMA labeled myofibroblastic transformation of hepatic stellate cells. The antibodies used for the immunohistological staining are mentioned in Table 1 including the applied dilutions and retrieval methods.

## 3. Results

### 3.1. Architecture: Generally Well Preserved

In all AL and DL samples, the overall lobular architecture was largely well preserved. A normal distribution pattern of portal tracts and central veins was recognizable. The transition from lesional to nonlesional tissue was usually recognizable by the slightly pushing, irregular border of the nonencapsulated tumor, except in hemorrhagic or necrotic parts where a fibrous scar may have developed and form a capsule. The regular transitional areas showed smaller, compactly arranged tumor hepatocytes to slightly larger hepatocytes of the nonlesional part, containing more cytoplasm. Portal tracts in these transitional areas frequently contained several thick-walled arteries but otherwise included normal bile ducts and portal veins. Portal inflammatory infiltrates varied but was usually nonconspicuous. Fibrosis was usually absent.

**Figure 1:**
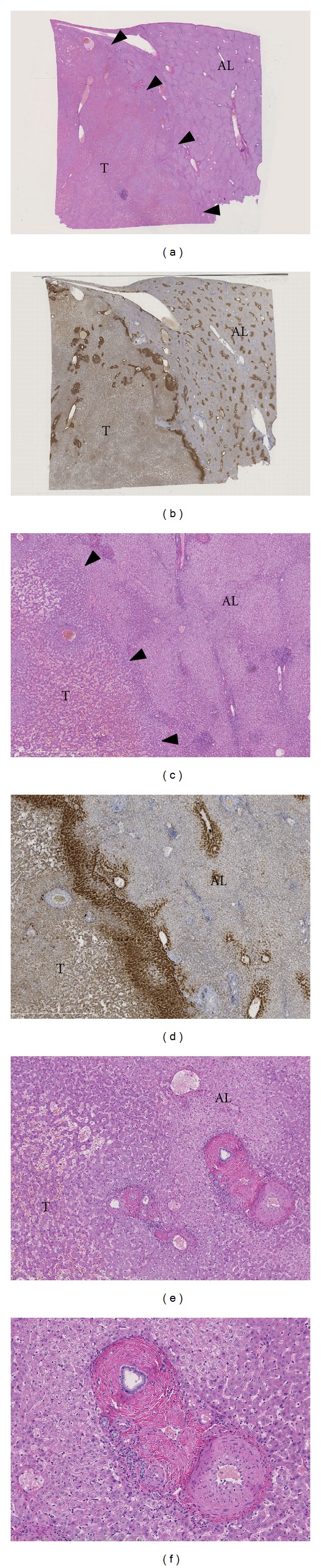
The transitional area of IHCA and adjacent liver. (a, c) HE stained whole slide (a) and detail (c) of a transitional area of an IHCA (T) and the nontumorous adjacent liver (AL). Arrowheads indicate the noncapsulated border of the tumor. (b, d) Glutamine synthetase expression of (a, c) highlights the difference in architecture of IHCA and adjacent liver (AL). In the AL part, glutamine synthetase expression in perivenular areas accentuates the preserved lobular architecture. (e, f) Portal tracts containing thick-walled arteries at the border of tumor (T) and adjacent liver (AL).

Figure 1 illustrates the several aspects of the transitional area.

### 3.2. Steatosis: Common Finding

Steatosis was a common finding in the nonlesional liver tissue as it was observed in 23/32 (70%) AL samples and 13/22 (59%) DL ones. The majority of cases showed mild to moderate degrees of steatosis. Severe steatosis is present in 3 AL and 2 DL samples. Steatohepatitis was rare, being present only in 2/32 patients, both in the AL and DL samples.

In the IHCA itself, steatosis was less common than in the nontumorous counterpart. Steatosis was present in 15/32 (47%) tumor samples. In the steatotic liver, based on the steatosis of AL samples, there was a similar frequency of IHCA with (12/23, 52%) and without fatty changes (11/23, 48%) whereas in the nonsteatotic liver most tumors were nonsteatotic (6/9, 67%). A steatotic tumor in a nonsteatotic liver is less common (3/9, 33%). When the frequencies were based on the steatosis of the DL samples, the majority of tumors in the steatotic liver contained fatty changes (8/13, 62%). Similar with the findings of the AL samples, tumors of nonsteatotic DL samples were mostly nonsteatotic as well (6/9, 67%). 

Of note, 20 of the 32 patients have high BMI values, leading to a median BMI of 32.55 ± 4.9.

**Figure 2:**
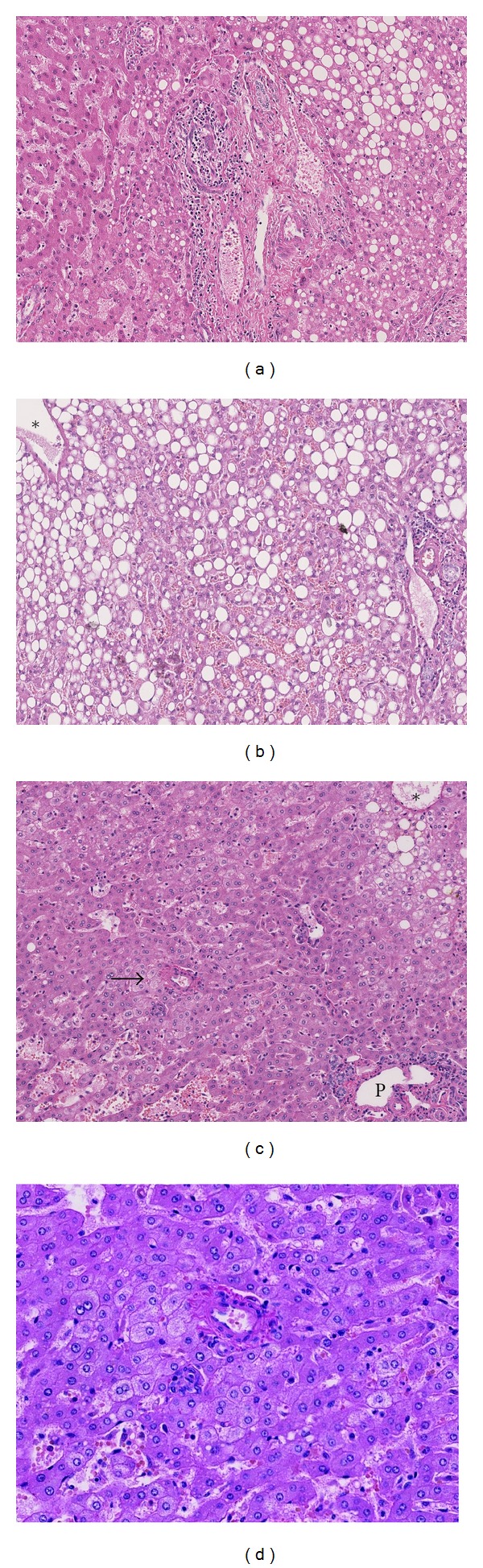
Steatosis and vascular changes. (a) The transitional area of a nonsteatotic adenoma (left part) with a steatotic adjacent nontumorous liver (right part). (b) Moderate steatosis in a distant sample. A portal tract is present in the right lower corner and a central vein in the left upper corner (*). (c) Vascular changes in a distant sample. A portal tract (P) is present in the right lower corner and a central vein in the right upper corner (*). An area with dilated sinusoids is present in the left lower corner. The arrow indicates a group of single arteries. (d) Detail of the single arteries.

Figures (2(a) and 2(b)) show the steatotic changes in the transitional area and in a DL sample.

### 3.3. Sinusoidal Dilatation: Frequent Phenomenon

SD was a frequent phenomenon in both AL and DL parts showing a frequency of 59% (19/32 cases) and 77% (17/22 cases), respectively. The areas of dilated sinusoids were of variable extent and rather randomly distributed in the lobules unlike the regular centrilobular punched-out pattern of outflow obstruction. Nevertheless, we have applied the Rubbia-Brandt et al. scoring system [13] that follows the lobular architecture to allow a semiquantitative scoring. In both AL and DL samples, the vast majority of SD was of mild degrees, as observed in 74% and 76% of those cases showing SD. Of the 5 cases with moderate and severe SD in their AL samples, 3 cases showed mild SD in their corresponding DL, 1 case had moderate SD, and 1 case had similarly severe SD in their DL. The 2 latter cases represented 2 of 4 DL cases with moderate and severe SD. The 2 remaining DL cases showed mild SD in their corresponding AL.

### 3.4. Single Arteries/Arterioles: Regularly Seen Even in Distant Samples

There was a similar frequency of single arteries/arterioles in AL and DL samples. Single arteries were present in 12/32 AL samples (38%) and in 8/22 DL samples (36%). As in the HCA, these single arteries were both present in small groups and as truly single arterial structures in the hepatic lobule (Figures 2(c) and 2(d)).

### 3.5. Immunohistology: Minute Foci of CRP-Positive Areas; Ubiquitous DR; Activated Myofibroblasts

The expression pattern of GS confirmed the preserved lobular architecture in AL and DL samples as shown by the perivenular distribution of cytoplasmic GS in hepatocytes in the centrilobular areas (Figures 1(a)–1(d)). Bile ductal and ductular cholangiocytes showed a faint blush of cytoplasmic GS expression. A normal membranous *β*-catenin labeling was present in all hepatocytes but no nuclear expression. Bile ducts and ductules also showed membranous but no nuclear *β*-catenin expression.

**Figure 3:**
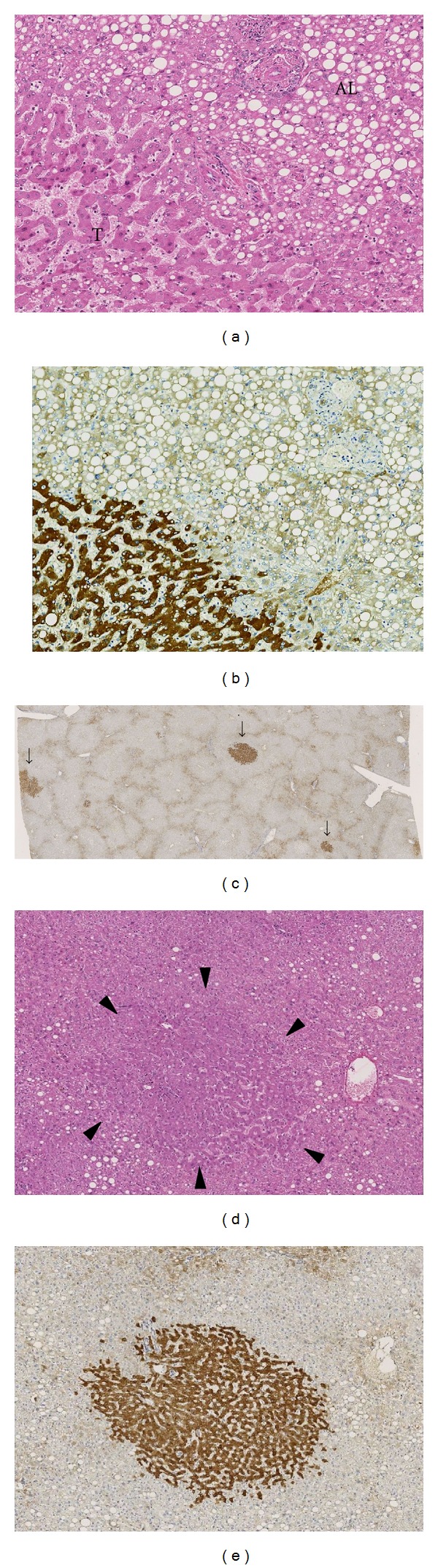
CRP expression in the transitional area and minute CRP-positive foci in a distant sample. (a) The transitional zone of an adenoma without steatosis (T) and steatotic adjacent liver (AL) in hematoxylin-eosin (HE) staining. (b) The same area as (a) in CRP immunostaining showing diffuse increase of CRP in the tumor part. (c) CRP immunostaining of a nontumorous liver sample distant from the tumor showing 3-minute CRP foci (arrows). The preserved architecture of the hexagonal liver lobules is highlighted by the vague expression of CRP which outlines the peripheral boundaries of the lobules. (d) Detail of a minute CRP-positive focus in HE showing an area in the lobule with slightly dilated sinusoids, more eosinophilic hepatocytes, and absence of steatosis which is present outside the contours of this focus (arrowheads). (e) The CRP expression of the focus described in (d).

All AL and DL samples showed a normal periportal pattern of CRP expression in hepatocytes. However, in 14/32 cases, minute foci of aberrant CRP expression were observed in the hepatic lobule, consisting of 6 AL samples, 4 DL samples, and 4 other cases of which both the AL and DL samples contained CRP-positive foci. Eleven of these 14 cases concerned livers with multiple adenomas (Figure 3). In the studied group, 21/32 IHCAs were multiple adenomas. None of the minute CRP-positive foci showed GS positivity and/or nuclear *β*-catenin expression.

Additional immunohistology was performed with K19, CD34, and *α*-SMA. 

**Figure 4:**
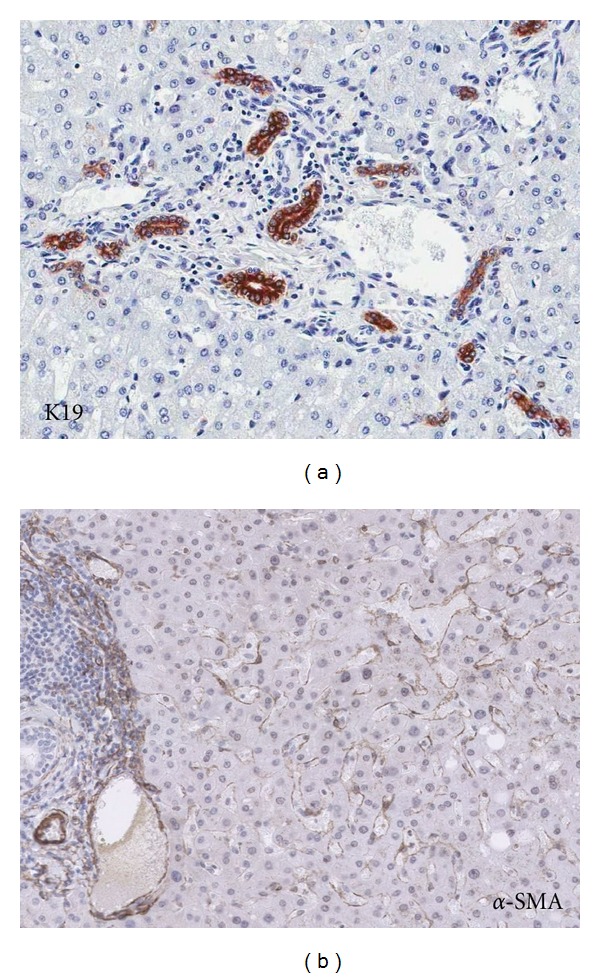
Ductular reaction and activated myofibroblasts in the nontumorous liver. (a) Ductular reaction in a portal tract of a distant nontumorous liver sample highlighted by K19 immunostaining. (b) Presence of *α*-SMA-positive myofibroblasts in a distant nontumorous liver sample, most obvious in dilated sinusoids.

DR was practically ubiquitous, being present in 29/32 (91%) AL samples and 21/22 DL (95%) ones (Figure 4(a)).

CD34 staining showed a normal distribution pattern of vascular endothelial labeling and periportal sinusoids. There was no increase of CD34 expression in the rest of the sinusoids. In contrast with CD34, an increased *α*-SMA expression in the sinusoids was seen in 28/32 (88%) AL and 17/22 DL (77%) (figure 4(b)). Diffuse increase of *α*-SMA was seen in 15/28 (54%) of the *α*-SMA-positive AL samples, and a focal increase localized in the areas of SD was present in 46%. A similar frequency was observed in the DL samples with 9/17 (53%) diffuse distribution and focal increase of *α*-SMA in 47% of the *α*-SMA-positive DL samples.

## 4. Discussion 

In contrast with HCC which usually develops in a liver with long-standing chronic liver disease, HCA is mostly found in an otherwise normal liver. IHCA is one of the variants of HCAs representing 40–50% of all HCAs [1]. The 2 different mutational backgrounds of IHCA concerning gp130 and STAT3 lead to a similar morphology and immunophenotype of increased SAA/CRP in the tumor hepatocytes [5]. 

In the present study, we analyzed the histological and immunophenotypical changes of the nonlesional liver parts of resected IHCA specimens which include samples taken adjacent to the tumor and distant ones. Similar types of histological and immunophenotypical features were found in these two sample types, albeit in variable degrees and frequencies. Among these changes some features represent changes that are also present in IHCA but otherwise not found in normal livers, for example, sinusoidal dilatation, single arteries, and foci of CRP-positive hepatocytes.

The lobular architecture is generally well preserved as also confirmed by the normal perivenular distribution pattern of GS expression. Steatosis is very common, being present in 60–70% of the distant and adjacent nonlesional samples, which is in accordance with the high BMI of our study population and in line with the reported relation of IHCA with obesity [7, 9]. Although the latter condition is known to enhance carcinogenesis [11], the tumorigenic role of obesity in IHCA has yet to be elucidated. In the steatotic livers, based on the pattern of the distant samples, almost two-thirds of the tumors contain fatty changes. In the nonsteatotic livers, two-thirds of the tumors are nonsteatotic. Although much higher numbers of patients are necessary for robust conclusions, the above findings indicate that fatty changes in the tumor might be secondary to the fatty constitution of the liver in which the IHCA develops. The scarcity of steatotic tumors in nonsteatotic livers supports this view. 

Of note are the vascular abnormalities consisting of sinusoidal dilatation and single arteries. Several types of vascular changes in the nontumorous liver have been described in an early study on telangiectatic focal nodular hyperplasia which is the obsolete term of IHCA according to the new classification [15]. These features are not found in normal livers, neither do these features belong to the spectrum of changes of fatty liver disease, but these features are characteristics of IHCA. Single arteries are also frequently found in HCC and are even part of the criteria to establish the diagnosis of early HCC [16]. Sinusoidal dilatation appeared to be a common finding in the nonlesional liver parts, both those adjacent to the tumor and the distant samples. The fact that sinusoidal dilatation is also present in the nonlesional tissue indicates a systemic effect. In our study group of women in their reproductive stage, long-term use of oral contraceptives may have a contributory role as these agents are known to cause sinusoidal dilatation and peliotic changes. However, the concurrent presence of sinusoidal dilatation and single arteries in the nonlesional liver parts is suggestive for a common background factor leading to these vascular abnormalities. In HCA, these changes have been related to an increased gene expression of Angiopoietin-1, a vascular growth factor of the Angiopoietin/Tie-2 system [17, 18]. Excess Ang-1 has been reported, both in animal models and *in vitro* to induce vascular remodeling including dilated sinusoids and vessel-forming capacity [19–21]. In another study, we have found increased Ang-1 in HCC [22] in which single arteries are frequently found. These arteries increase in numbers paralleling tumor growth in HCC, and this phenomenon is regarded as tumor angiogenesis as the arteries form the vascular supply of the growing tumor [23]. The fact that there is no obvious tumor growth in the nonlesional liver of our IHCA study group renders angiogenic activity in these parts rather redundant. It is however plausible that an excess of Ang-1 produced by the tumor, may exert its effects in the nonlesional parts. In particular, because Tie-2 receptor which is the specific tyrosine kinase receptor of Ang-1, is ubiquitously present on the sinusoidal endothelial cells and vascular endothelial cells of histologically normal livers [17]. 

The dilated sinusoids usually lead to variable degrees of atrophy of the hepatic parenchyma. Paralleling these degenerative changes is the increased expression of *α*-SMA in these areas, reflecting activation of hepatic stellate cells into myofibroblasts. The latter process is probably also induced by steatosis which is present in the majority of cases and which is known to be a potent inducer of myofibroblastic activation. Variable types of hepatocellular damage are apparently present in the nonlesional liver of IHCA which would require replenishment of cellular loss. The presence of ductular reaction in nearly all samples reflects the regenerative activity. Ductular reaction has been described in fatty liver disease [24], but in general, it reflects a reparative activity that includes several progenitor cell niches [14].

Apart from the degenerative changes, the findings of CRP-positive foci outside the tumor and within liver tissue with preserved lobular architecture are most intriguing. It is tempting to speculate that those foci may represent minute HCA, particularly because most of those foci were found in cases with multiple adenomas. Our findings largely confirm the results of Bioulac-Sage et al. who also found additional CRP-positive micronodules in multiple IHCA, measuring between 2 to 10 mm, and containing features of IHCA [25]. These micronodules are mostly slightly larger than the CRP foci in the present study which are mostly smaller than 2 mm and in the majority of cases were found in random samples. Due to its subtlety, these foci are easily overlooked, first during gross examination of the resected specimen and secondly in routine HE staining. This may probably lead to the reported absence of these foci in many other cases of multiple IHCA. To avoid this sampling error it is recommendable to investigate the nonlesional liver tissue more robustly, for example, to include more sampling and application of additional CRP staining. If positive foci are found, it may indicate the presence of minute foci of HCA in the remnant liver which may have consequences for the followup management. The long-term behavior of small HCA foci and under specific circumstances such as pregnancy is not fully established. A recent study on the management of HCA during pregnancy has shown that discouragement of pregnancy in certain cases is no longer necessary because close monitoring of patients with small adenomas seems to offer adequate surveillance [26]. Whether this applies to multiple adenomas with multiple CRP positive foci is yet unclear.

In conclusion, from the architectural point of view, the nonlesional liver part of IHCA may be considered normal. However, the CRP-positive foci indicate that in cases with multiple adenomas, minute foci of adenomas may be present, also in the remnant liver. The presence of vascular abnormalities beyond the tumor and beyond the CRP foci needs further study, especially due to the similarities with the changes in the HCA. The high incidence of steatosis does not only confirm the hepatic manifestation of obesity in this group of patients. It provides another evidence that the normal liver in IHCA does contain abnormalities and in selected cases should probably be considered as a diseased liver. 

## Figures and Tables

**Table 1:** Antibodies applied for immunohistology.

Antibody	Dilution	Retrieval methods	Company
*β*-catenin	1 : 100	Tris-EDTA	BD Transduction (USA)
GS	1 : 4000	Tris-EDTA	Millipore (USA)
CRP	1 : 200	Tris-EDTA	Abcam (UK)
SAA	1 : 200	Protease 8 min	Dako (DK)
CK19	1 : 100	Protease 12 min	BD Bioscience (USA)
CD34	1 : 20	Tris-EDTA	Dako (DK)
α-SMA	1 : 800	Tris-EDTA	Dako (DK)
